# Efficient Lewis Acid Ionic Liquid-Catalyzed Synthesis of the Key Intermediate of Coenzyme Q_10_ under Microwave Irradiation

**DOI:** 10.3390/molecules15129486

**Published:** 2010-12-22

**Authors:** Yue Chen, Yuangang Zu, Yujie Fu, Xuan Zhang, Ping Yu, Guoyong Sun, Thomas Efferth

**Affiliations:** 1 Key Laboratory of Forest Plant Ecology, Ministry of Education, Northeast Forestry University, Harbin 150040, China; E-Mails: chenyuezds@yahoo.com.cn (Y.C.); 350330842@163.com (X.Z.); yup2010@163.com (P.Y.); shikong120@yahoo.com.cn(G.S.); 2 Engineering Research Center of Forest Bio-preparation, Ministry of Education, Northeast Forestry University, Harbin 150040, China; 3 Department of Pharmaceutical Biology, Institute of Pharmacy, University of Mainz, 55099 Mainz, Germany; E-Mail: t.efferth@dkfz.de (T.E.)

**Keywords:** ionic liquids, FT-IR, acidity, microwave irradiation

## Abstract

An efficient synthesis of a valuable intermediate of coenzyme Q_10_ by microwave-assisted Lewis acidic ionic liquid (IL)-catalyzed Friedel-Crafts alkylation is reported. The acidity of six [Etpy]BF_4_-based ionic liquids was characterized by means of the FT-IR technique using acetonitrile as a molecular probe. The catalytic activities of these ionic liquids were correlated with their Lewis acidity. With increasing Lewis acid strength of the ionic liquids, their catalytic activity in the Friedel-Crafts reaction increased, except for [Etpy]BF_4_-AlCl_3_. The effects of the reaction system, the molar fraction of Lewis acid in the Lewis acid ILs and heating techniques were also investigated. Among the six Lewis acid ionic liquids tested [Etpy]BF_4_-ZnCl_2_ showed the best catalytic activity, with a yield of 89% after a very short reaction time (150 seconds). This procedure has the advantages of higher efficiency, better reusability of ILs, energy conservation and eco-friendliness. The method has practical value for preparation of CoQ_10_ on an industrial scale.

## 1. Introduction

**Scheme 1:**
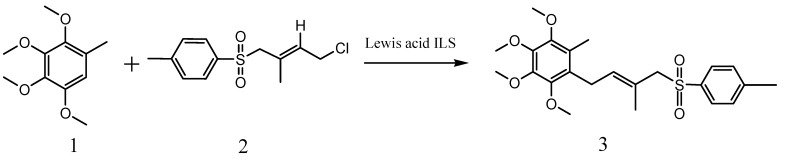
The Friedel–Crafts reaction for the synthesis of compound **3**.

Isoprenoid natural products containing a *p*-quinone groups play an important role in biological processes and display many interesting biological properties including anticancer, anti-oxidative, antifungal, antiviral, and anti-inflammatory activities [1,2,3]. For example, coenzyme Q_10_, also known as ubiquinone, is widely used as a drug against many diseases [4,5]. Although many methods for the synthesis of this indispensable natural product have been described [6,7,8], the key step is the direct coupling method of the quinone core and the prenyl side chain by a Friedel–Crafts (F-C) reaction to afford the key intermediate 1-[(*E*)-3-methyl-4-benzenesulfonyl-3-methylbut-2-enyl]-2,3,4,5-tetra-methoxy-6-methylbenzene (**3**, Scheme 1) [9]. However, this reaction has always suffered from low yields, long reaction times, high temperatures, use of solvents hazardous to the environment and so on. In order to overcome these disadvantages and to meet the considerable market demands for the end product, a new more efficient and eco-friendly synthesis method of compound **3** is desirable.

Ionic liquids (ILs) have attracted growing academic and industrial interest because of their special properties including excellent thermal and chemical stability, low vapor pressure, good tunable solubility and reusability [10,11,12]. Especially in F-C alkylation reactions, ionic liquids have been applied successfully as a way of avoiding corrosive and polluting catalyst systems such as HF or AlCl_3_ in chloroform or benzene [13,14]. Although numerous investigations on the application of ILs in alkylation reactions have been carried out, studies of the acidity–activity relationships of Lewis acid ionic liquids are still rather rare [15,16]. 

In addition, microwave–assisted synthesis has attracted much attention in recent years in promoting efficiency, decreasing reaction times and saving energy in many organic reactions [17,18,19,20]. The strong polar nature of ILs makes them an ideal reaction medium under microwave irradiation [21,22,23]. However, to our knowledge, the combined use of ionic liquids and microwave technology to accomplish the synthesis of intermediate **3** of coenzyme Q_10_ has not been reported as yet.

**Figure 1:**
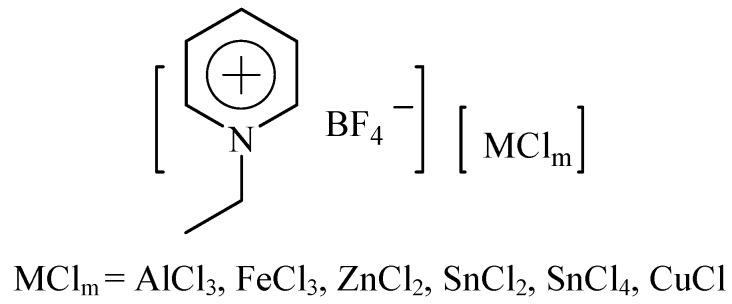
The structure of[Etpy]BF_4_-MCl_m_ ionic liquids.

Herein, we report the solvent-free synthesis of compound **3** using various [Etpy]BF_4_-based Lewis acid ILs under microwave irradiation (Figure 1). We focused on the efficient application of Lewis acid ILs in F-C alkylation reactions and demonstrated the acidity-activity relationship of [Etpy]BF_4_-based Lewis acidic ILs in these reactions. We used acetonitrile as a molecular probe to measure the Lewis acidity of different [Etpy]BF_4_-based ILs by means of IR spectroscopy. The influence of different reaction conditions was also investigated. Lewis acid ILs play an important role in avoiding the use of toxic or flammable organic solvents, and microwave irradiation can significantly enhance the reaction rates. This procedure, followed by an eco-friendly and simple workup procedure, gave the corresponding products in good yields.

## 2. Results and Discussion

### 2.1. FT-IR characterization of Lewis acid ionic liquids

In order to study the relationship of acidity and activity of Lewis acid ILs, their acidities need to be characterized first. Several techniques, for example UV, NMR and electrochemical methods, have been used to monitor the interactions between donors and anionic species [24,25,26]. However, all of these techniques have disadvantages such as strong medium-specificity, little available reference information and/or difficulties in manipulation. We used a method for measuring the Lewis acidity as described by Yang [27]. Acetonitrile, a weak base, is used as a base probe molecule to determine the acidity of ionic liquids. As a result, the IR absorption bands of C≡N are weakened and shift in the blue direction [27]. This method is easy to perform and an efficient way to characterize the acidity of ILs.

**Figure 2:**
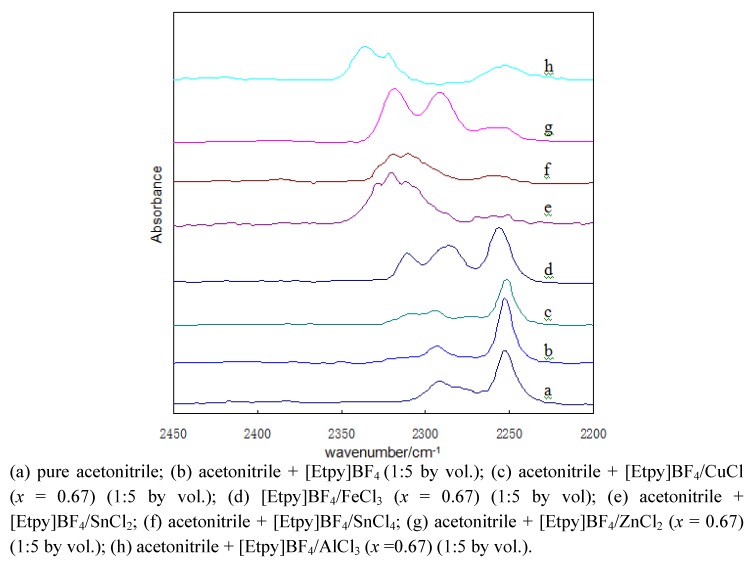
FT-IR spectra of ionic liquid using acetonitrile as IR probe.

As shown in Figure 2, two IR bands at 2,252 and 2,292 cm^−1^, due to C≡N stretching vibrations, can be observed for pure acetonitrile (Figure 2a). When mixed with the ionic liquid [Etpy]BF_4_, these two IR bands were essentially unchanged, and no new band was observed (Figure 2b), which implies that [Etpy]BF_4_ has no Lewis acidity. However, the addition of acetonitrile to [Etpy]BF_4_-MCl_m_ (with the apparent mole fraction of MCl_m_, x, is 0.67 in each case) results in the appearance of a new bands at higher wave numbers occurring at 2,308 cm^−1^ for [Etpy]BF_4_-CuCl, 2,310 cm^−1^ for [Etpy]BF_4_-FeCl_3_, 2,320 cm^−1^ for [Etpy]BF_4_-SnCl_2_, 2,320 cm^−1^ for [Etpy]BF_4_-SnCl_4_, 2,319 cm^−1^ for [Etpy]BF_4_-ZnCl_2_, and 2,337 cm^−1^ for [Etpy]BF_4_-AlCl_3_, respectively (Figures 1c–h) . The stronger Lewis acid ILs led to a stronger Lewis acid–base interaction between the ionic liquid and acetonitrile [28], and therefore, the C≡N stretching region shifts to higher wave numbers so based on the wave numbers of the blue shift of the new IR band for [Etpy]BF_4_-based ILs their Lewis acidity increased in the following order: [Etpy]BF_4_-AlCl_3 _> [Etpy]BF_4_-ZnCl_2_ > [Etpy]BF_4_-FeCl_3 _> [Etpy]BF_4_-CuCl. Additionally, the Lewis acidity of [Etpy]BF_4_-ZnCl_2_ was similar to that of [Etpy]BF_4_-SnCl_2_ and [Etpy]BF_4_-SnCl_4_. From the intensity of the new IR band for [Etpy]BF_4_-based ILs, it was noted that the intensity of the new bands of [Etpy]BF_4_-AlCl_3_, [Etpy]BF_4_-ZnCl_2_, [Etpy]BF_4_-SnCl_2_, and [Etpy]BF_4_-SnCl_4_ were greater than those of the [Etpy]BF_4_-FeCl_3_ and [Etpy]BF_4_-CuCl, also indicating that number of acid sites on the former was higher.

### 2.2. Comparison of the Friedel–Crafts reaction in organic solvents with in ionic liquids

We chose two moderate Lewis acid ILs ([Etpy]BF_4_-FeCl_3_, [Etpy]BF_4_-ZnCl_2_) as models to investigate the influence of normal organic solvents and ILs on the reaction. Table 1 summarizes the results of a comparative study of synthesis of compound **3** in [Etpy]BF_4_-FeCl_3_, [Etpy]BF_4_-ZnCl_2_ and in organic solvent systems, respectively. 

**Table 1:** Comparative results of Friedel–Crafts reactions in different solvent.

Entry	Catalyst/Solvent	Reaction Temperature(°C )	Reaction Time (hrs)	Yield ^c ^(%)
1	FeCl_3_/C_2_H_2_Cl_2 _^a^	80	7	51
2	ZnCl_2_/C_2_H_2_Cl_2_^ a^	80	8	75
3	[Etpy]BF_4_-FeCl_3_^b^	60	2	60
4	[Etpy]BF_4_-ZnCl_2_^ b^	60	2	89

^a^ Reaction conditions: *n*(MCl_m_):*n*(compound 1) = 1.5:1, *n*(compound 1):*n*(compound 2) = 1:1.2, the mixture of reactants was stired in the presence of MCl_m_ (FeCl_3_ or ZnCl_2_) at 80 °C; ^b^ Reaction conditions: *n*(MCl_m_/[Etpy]BF_4_):*n*(compound 1) = 1.5:1, *n*(compound 1):*n*(compound 2) = 1:1.2. The molar fraction of MCl_m _in ILs was 0.67 and the mixture of reactants was stirred at 60 °C;^c^ Isolated yield.

In organic solvents, the F–C alkylations catalyzed by simple Lewis acids required longer reaction times (up to 7 hrs) and gave lower yields (entries 1–2). Compared with the organic solvent systems, the reactions performed in [Etpy]BF_4_-based Lewis acid ILs produced the corresponding products in the shorter times and higher yields (entries 3–4). [Etpy]BF_4_-ZnCl_2_ showed outstanding catalytic activity, with a high yield (89%) in shorter time (2 hrs). Furthermore, in the presence of [Etpy]BF_4_-based Lewis acid ILs, the isolation and purification for the target products was much easier and the alkylation reaction mixture was free of any volatile organic solvent. [Etpy]BF_4_-ZnCl_2_ appeared to be a suitable dual functional Lewis acid catalyst and solvent for F–C alkylation reactions.

### 2.3. Effect of molar fraction of Lewis acid in ionic liquid on the reaction yields

The influence of varying molar fractions of Lewis acid [*x*(MCl_m_)] in the ionic liquid catalyst on the alkylation was studied next (Table 2). The molar fractions of ZnCl_2_ and FeCl_3_ in [Etpy]BF_4_-base ionic liquid had a crucial influence on the reactions. The yield of the product changed with various molar fractions of *x* (MCl_m_) from 0.33 to 0.75 under identical reaction conditions. The ionic liquid exhibited no catalytic activity, when* x*(FeCl_3_) was 0.5 and *x*(ZnCl_2_) was 0.33 (entry 1, 2). Because ILs are stoichiometrically neutral, the reaction yields remarkably increased with an increase of *x* from 0.5 to 0.67 (entry 2, 3, 4). The yield of target product was highest, when the molar fraction of MCl_m_(*x*) was 0.67 (entry 4), while the yield decreased when *x* > 0.67 due to promoting undesirable side reactions. As has been discussed, ILs are basic, when the molar fraction of most Lewis acids (FeCl_3_, AlCl_3_) in ILs are less than 0.5 (ZnCl_2_ is 0.33). Above this point, however, the ILs would be acidic [25,29]. With increasing of the molar percentage of Lewis acid, acidity of ILs became stronger [30]. Since F–C reactions are promoted by Lewis acid catalysts, the elevated acidic strength will improve the reaction yield. Meanwhile, it should be noted that excess Lewis acidity of ILs appeared to be detrimental to the reaction presented here. This phenomenon implies that Lewis acid ILs had the best suitable acid strength when the molar fraction of Lewis acid in Lewis acid ILs was 0.67. This result was in accord with other reports [28].

**Table 2:** Effect of molar fraction of Lewis acid in ionic liquid on the Friedel–Crafts reaction^a^.

Entry	*x*(ZnCl_2_)	*n*(ZnCl_2_):*n*([Etpy]BF_4_)	Yield ^b^ (%)	
			ZnCl_2_	FeCl_3_
1	0.33	0.5:1.0	0	0
2	0.50	1.0:1.0	51	0
3	0.60	1.5:1.0	75	50
4	0.67	2.0:1.0	89	60
5	0.71	2.5:1.0	86	58

^a^ Reaction conditions: *n* (ZnCl_2_/[Etpy]BF_4_):*n*(compound1) = 1.5:1, *n*(compound 1):*n*(compound 2) = 1:1.2, the mixture of reactants was stired at 60 °C for 2 hrs; ^b^ Isolated yield.

### 2.4. Catalytic activities of various Lewis acid ionic liquids

Further, we studied the influence of various ILs of different acidic strengths on the reaction (Table 3). Pure [Etpy]BF_4_ did not reveal any catalytic activity (entry 1). [Etpy]BF_4_-CuCl also showed no catalytic activity, even after prolonged time (entry 2), probably because it is too weakly acidic, as can be seen from Figure 2. [Etpy]BF_4_-FeCl_3_ exhibited moderate results, and a yield of 60% was obtained after 2 hrs (entry 3); [Etpy]BF_4_-ZnCl_2 _showed the best catalytic activity with a high yield (89%) (entry 6). The catalytic effects of [Etpy]BF_4_-SnCl_2 _and [Etpy]BF_4_-SnCl_4 _approached that of [Etpy]BF_4_–ZnCl_2_, giving 85% and 86% yields, respectively (entries 4,5). The catalytic activity in the F-C reaction increased with increasing acidity strength of Lewis acid ILs. However, the reaction time in [Etpy]BF_4_- SnCl_2 _and [Etpy]BF_4_-SnCl_4_ catalyzed reactions were both longer than with [Etpy]BF_4_-ZnCl_2__, _because the catalytic performance of Lewis acid ILs in F-C alkylation was not only related to the acidic strength, but was also affected by the metal ions. In addition, other side reactions occurred and the corresponding product was not observed when using [Etpy]BF_4_-AlCl_3 _as catalyst (entry 7); the reaction product mixture was complex and there was no main product, probably owing to its too high acid strength and/or sensitivity to moisture. To summarize, the suitable acidity of Lewis acid ILs may play an important role in the order of alkylation capability. In our experience, [Etpy]BF_4_–ZnCl_2_ was the best catalytic system for the F-C alkylation reaction to synthesize compound **3**. Our results indicate that the catalytic capability of [Etpy]BF_4_-based Lewis acid ILs for this F-C alkylation is essentially correlated with the acid strength of the catalytic procedure.

**Table 3:** Catalytic activities of Friedel–Crafts alkylation reaction with various ionic liquids ^a^.

Entry	Ionic Liquid	Reaction Time (hrs)	Yield ^c^ (%)
1	[Etpy]BF_4_	20	0
2	[Etpy]BF_4_-CuCl	20	0
3	[Etpy]BF_4_-FeCl_3_	2	60
4	[Etpy]BF_4_-SnCl_2_	8	85
5	[Etpy]BF_4_-SnCl_4_	4	86
6	[Etpy]BF_4_-ZnCl_2_	2	89
7	[Etpy]BF_4_-AlCl_3_^b^	1	0

^a^ Reaction conditions: *n*(MCl_m_/[Etpy]BF_4_):*n*(compound 1) = 1.5:1, *n*(compound 1):*n*(compound 2) = 1:1.2, the molar fraction of MCl_m _in ILs is 0.67, the mixture of reactants was stirred at 60 °C in oil heating; ^b^The reaction system was complex and there were not the corresponding product and other main products; ^c^ Isolated yield.

### 2.5. Catalytic activities of various ionic liquids in microwave heating

In the microwave irradiation method, the substrates were reacted in the presence of different Lewis acid IL catalysts at 60 °C (Table 4.). The reactions performed using microwave heating gave the target product in considerably shorter times (150 s~180 s) than by means of conventional heating (2 hr~8 hr; see Table 3), although the yields did not change significantly. Thus, the influence of various ILs on the reaction yields obtained by microwave heating was in accord with the ones obtained by conventional heating. [Etpy]BF_4_-ZnCl_2_ showed the best catalytic activity, providing the highest yield of the desired compound (89%) after a shorter reaction time (150s). Microwave-enhanced chemistry is based on the efficient heating of materials by “microwave dielectric heating” effects. Here, the energy can be transferred to the reaction media by two mechanisms - dipole rotation and ionic conduction [31]. The rates of reactions involving polar components are usually very fast in microwave irradiation. The high polarity of the IL makes it an efficient reaction medium for rapid heating under microwave irradiation [32]. Our experiments indicated that reaction mixtures exposed to microwaves allowed an easy and rapid reaction to obtain compound **3** by F-C reactions in Lewis acid ILs. The result was in agreement with the mechanism of microwave heating.

**Table 4:** Catalytic activities of alkylation reactions with various ionic liquids in microwave heating ^a^.

Entry	Catalyst/Solvent	Reaction Time (s)	Yield ^b^(%)
1	[Etpy]BF_4_-FeCl_3_	150	60
2	[Etpy]BF_4_-SnCl_2_	180	85
3	[Etpy]BF_4_-SnCl_4_	180	84
4	[Etpy]BF_4_-ZnCl_2_	150	89

^a^ Reaction conditions: n(MClm/[Etpy]BF4):n(compound 1) = 1.5:1, n(compound 1):n(compound 2) = 1:1.2. The molar fraction of MClm in ILs was 0.67 and the mixture of reactants was stirred at 60 °C by 300W microwave heating; ^b^ Isolated yield.

### 2.6. Recycling and reuse of [Etpy]BF4-ZnCl2

**Figure 3:**
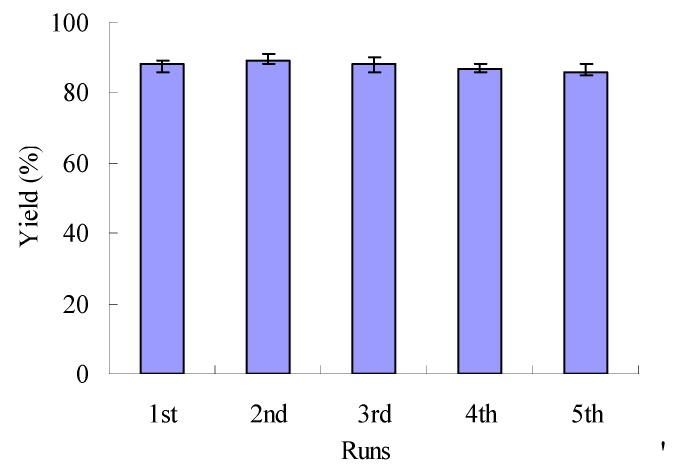
Recyclability of the [Etpy]BF_4_-ZnCl_2_ catalyst.

Recycling of Lewis acid ILs is clearly important in the context of economic feasibility. In view of the efficiency of the reaction in [Etpy]BF_4_-ZnCl_2_, we examined its recyclability. Once product had been extracted as described in the Experimental, the IL was evaporated under vacuum. [Etpy]BF_4_-ZnCl_2_ was reused at the same reaction after extracting out the organic phase under vacuum and drying at 80–100 °C for 30 min. These results are shown in Figure 3. The yield was only slightly decreased after the fifth cycle, indicating that the catalyst possessed an excellent reusability under the same reaction conditions.

## 3. Experimental

### 3.1. General

All analytical grade chemicals were purchased from the Shanghai Chemical Reagent Co. Ltd. and used without further purification. The IR spectra were recorded on a Shimadzu IR Affinity-1 FTIR spectrophotometer at room temperature. ^1^H-NMR analyses were run in CDCl_3 _on a Bruker-300 instrument with TMS as internal standard. MS analysis was carried out on an API-3000 LC-MS- MS instrument.

### 3.2. Preparation and FT-IR characterization of Lewis acid ionic liquids

[Etpy]BF_4_–AlCl_3_, [Etpy]BF_4_–FeCl_3_, [Etpy]BF_4_–ZnCl_2_, [Etpy]BF_4_–CuCl, [Etpy]BF_4_–SnCl_2_ and [Etpy]BF_4_–SnCl_4_ were prepared by the reported procedure [16]. The appropriate anhydrous Lewis acid salt was slowly added to a solution of [Etpy]BF_4_, and the mixture was stirred at 65 °C until the Lewis acid was completely dissolved to give the [Etpy]BF_4_–MCl_m_ ionic liquids. FT-IR characterization of ionic liquids was performed by using acetonitrile at a specific volume ratio (acetonitrile to ionic liquids = 1:5) as a molecular probe, and spreading the samples on a KBr wafer.

### 3.3. Synthesis of 1-[(E)-3-methyl-4-benzenesulfonyl-3-methylbut-2-enyl]-2,3,4,5-tetra-methoxy-6-methylbenzene *(**3**)* and analysis

Reactants were directly added to a solution of [Etpy]BF_4_-MCl_m_ILs (with the apparent mole fraction of MCl_m_, *x* is 0.67 in each case). Then stirring was started at a desired temperature achieved by means of conventional heating or microwave irradiation. After the reaction ended, the reaction mixture was extracted with warm (about 40~50 °C) chloroform and the reaction mixture became two liquid phases, an organic phase and an ionic liquid phase. The product was extracted in the organic phase, and then the product was purified by crystallization. The ionic liquid phase was evaporated, dried in vacuum and reused again. Data for compound **3** are: ^1^H-NMR: (ppm): 1.94 (3H, s, CH_3_), 1.98 (3H, s, CH_3_), 2.40 (3H, s, CH_3_), 3.24 (2H, d, CH_2_), 3.70–3.91 (12H, 4s, 4 × OCH_3_), 3.69 (2H, s, -CH_2_-SO_2_), 4.91 (1H, t, -CH=), 7.21–7.24 (2H, d, *J* = 9.0 Hz, Hb-Ph), 7.64–7.67 (2H, d, *J* = 9.0 Hz, Ha-Ph). MS (ESI): [M+H]^+^435.3, [M+Na]^+^457.1. IR/cm^1^: 2,933, 1,470, 1,406, 1,352, 1,312, 1,162, 1,132, 1,109, 1,087, 1,069, 1,040, 965, 892, 742, 623, 581, 532, 510.

## 4. Conclusions

In the present paper, we have developed a Lewis acid IL-catalyzed synthesis of compound **3**, the key intermediate in the synthesis of Co-Q_10_, by means of microwave irradiation. The relationships between acidity and catalytic activity of six [Etpy]BF_4_-based Lewis acid ILs were studied. Their Lewis acidity was characterized by means of IR using acetonitrile as a molecular probe. We demonstrated that the catalytic behavior of these Lewis acid ILs was correlated with their Lewis acidity strength. Among the six Lewis acid ILs tested, [Etpy]BF_4_-ZnCl_2_ showed outstanding catalytic activity in this F‑C reaction. The reactions provided the products in higher yields and in much shorter reaction times than reported in the previous literature. Our method incorporates the dual advantages of IL catalysis and the use of microwave irradiation. Thus, the procedure is effective, eco-friendly and requires lower energy input. The isolation of the product is easy, and the IL catalyst has an excellent reusability. This method should prove valuable for the industrial synthesis of CoQ_10_.
